# Isolation and Identification of the Sex Pheromone of *Evergestis extimalis* Scopoli (Lepidoptera: Pyralidae)

**DOI:** 10.3390/insects17010064

**Published:** 2026-01-05

**Authors:** Mingang Qin, Youhua Ma, Youpeng Lai, Siyu Liu, Gui Zhang

**Affiliations:** Key Laboratory of Integrated Pest Management of Qinghai Province, Qinghai University, Xining 810016, China; 18189603228@163.com (M.Q.); m2218937@163.com (Y.M.);

**Keywords:** insect pest of spring rape, GC-EAD and GC-MS, E11-14Ac, field attracting test

## Abstract

In Qinghai Province, *Evergestis extimalis* is an important pest of spring rape and is primarily controlled using chemical pesticides. Sex pheromones offer an alternative method for pest management in relatively non-polluted environments. In this study, the sex of *E. extimalis* pupae was identified, followed by isolation and structural characterization of female pheromone components using GC-EAD and GC-MS. A field attraction assay was then performed. The results showed that female pupae possess a longitudinal crack on the upper central ventral surface of the eighth abdominal segment, which connects the seventh and ninth abdominal segments. The two sides of this crack are open and flat, without protruding semicircles. Male pupae lack this longitudinal crack on the eighth abdominal segment but display one on the central ninth ventral segment, accompanied by semicircular tubercles on each side. The primary component extracted from female sex glands was identified as E11-14Ac. In field trials, E11-14 displayed a stronger attractive effect on *E. extimalis* males than the other tested attractants. In conclusion, E11-14Ac was preliminarily identified as the main component of the sex pheromone of *E. extimalis*, providing a foundation for its control using sex pheromones.

## Simple Summary

*Evergestis extimalis* is a major destructive pest in spring rape fields on the Qinghai Plateau in China. At present, chemical pesticides are widely applied in agricultural production to control this species. To protect the agricultural and pastoral ecosystems of the Qinghai–Tibet Plateau—known as the world’s third pole—and to reduce pesticide usage, we identified the sex pheromone of this pest, developed pheromone lures, and preliminarily assessed their trapping effectiveness in field trials. This study provides a basis for the biological and ecological management of this pest.

## 1. Introduction

Insect pheromones are chemical substances that play essential roles in communication related to foraging, courtship, spawning, alarm signaling, and defense. Different types of pheromones include alarm, aggregation, epideictic, sex, territory, and trail pheromones [1]. Insect sex pheromones are trace chemicals released by exocrine glands that stimulate courtship behavior and provoke corresponding physiological responses in opposite-sex individuals. Currently, the Pherobase lists pheromones and semiochemicals of more than 7500 species and 6500 semiochemical compounds [2], with research covering 9 orders and 90 families [1]. Sex pheromones of more than 700 moth species have been identified [3]. In Lepidoptera, sex pheromones are secreted from specific sites on the eighth or ninth abdominal segment [4]. Courtship behavior appears during reproductive maturity, at which time insects release sex pheromones that attract opposite-sex individuals and initiate mating behavior [4,5]. Typically, the peak courtship period coincides with peak pheromone release. Based on chemical structure, insect sex pheromones are divided into four types: Type I, Type II, Type III, and Type 0 [3]. Type I pheromones contain long C10–C18 straight-chain, unsaturated alcohols, aldehydes, and acetates synthesized from hexadecanoic and octadecanoic acids through multistep biochemical pathways in the pheromone gland. Type I pheromones account for approximately 75% of known Lepidoptera pheromones in species such as *Spodoptera frugiperda* [6], *Spodoptera litura* [7], *Helicoverpa armigera* [8], and *Phthorimaea operculella* [9].

Insect pheromone extraction methods include the organic solvent immersion method, solid-phase microextraction, and dynamic headspace adsorption. Candidate pheromone compounds are first screened using electrophysiological responses, after which GC-MS or GC × GC-TOF-MS is used for structural identification. Retention times and mass spectra are then compared with authentic standards to confirm compound identity [10].

Sex pheromones play important roles in male attraction and mating disruption and are useful tools for pest monitoring and quarantine. When incorporated into lures and trapping systems, they can suppress pest populations on a large scale. Mating disruption techniques hinder male access to females, thereby preventing or delaying mating, reducing pest populations, and lowering crop damage [11].

In Qinghai Province, *Evergestis extimalis* Scopoli is a major pest in spring rape fields. Chemical pesticides remain the primary control strategy. However, prolonged application of high pesticide doses has led to resistance development [12] and potential environmental contamination. Because pheromone-based trapping is environmentally benign, it is well-suited for integrated pest management. Identifying the sex pheromones of *E. extimalis* will support subsequent research, production, and practical application.

## 2. Materials and Methods

### 2.1. Agents and Equipment

Artificial Climate Chamber (Model: SRG-520A-3LED, Hangzhou Shuolian Instrument Co., Ltd., Hangzhou, China).

Dissecting microscope (Model: Stemi 508, Carl Zeiss Microscopy GmbH, Jena, Germany). Refrigerator (Model: BCD-29W, Qingdao Haier Co., Ltd., Qingdao, China).

GC-EAD (Gas chromatography–electroantennogram detection) (Model: Agilent 7820A–Syntech, Agilent Technologies, Santa Clara, CA, USA; Syntech, Kirchzarten, Germany).

GC-MS (Gas chromatography–mass spectrometry) (Model: Agilent 7820A–5977B, Agilent Technologies, Santa Clara, CA, USA).

E11-14Ac, Z11-14Ac, 7Z-12Ac, 9Z-12Ac (Shanghai Udchem Agricultural Technology Co., Ltd., Shanghai, China).

Hexane (analytical grade), Vaseline, 1 mL centrifuge tubes, disposable lunch boxes, disposable plastic cups, Petri dishes, and tweezers. 

### 2.2. Insect

Larval cocoons of *E. extimalis* collected from fields (Datong County, Xining City; 37°0′24″ N, 101°38′24″ E) were maintained at 0 °C for 55–60 days, corresponding to the pre-pupal stage following overwintering. Subsequently, the pupal cocoons were placed individually in Petri dishes (35 mm × 10 mm) and reared in an artificial climate chamber at 20 ± 1 °C, 60 ± 5% RH, and a photoperiod of L:D = 14:10 h.

### 2.3. Methods

#### 2.3.1. Distinguishing the Sex of *E. extimalis*

Sex identification in *E. extimalis* pupae was based on morphological characteristics of the abdominal end [13]. Identified pupae were reared individually. The pupation process of larvae was monitored daily until completion. Adult morphological characteristics were recorded after eclosion.

Additionally, approximately 30 pairs of female and male pupae were paired and reared until adult emergence. Sex identification accuracy was assessed based on successful mating and egg laying.

#### 2.3.2. Extraction of Sex Pheromone

Female adults were used for pheromone extraction, whereas males were used for EAD analysis. The extraction procedure followed Huang et al. [14]. The peak courtship period was determined to identify the optimal extraction time.

A total of 20 virgin females (1–3 days after eclosion) were selected. Gentle pressure on the abdomen induced ovipositor extrusion. Three terminal abdominal segments were removed to excise the sex gland. Five sex glands were immersed in 100 μL hexane for 40 min, after which the glands were discarded. Extracts were stored at −20 °C prior to further analysis.

#### 2.3.3. GC-EAD Analysis of Sex Gland Extracts

Gland extracts from female *E. extimalis* were analyzed using GC-EAD according to Jiang et al. [15] with minor modifications. An Agilent 7820A GC equipped with a flame ionization detector (FID) and an HP-5 capillary column (30 m × 0.25 mm ID, 0.25 μm film; J&W Scientific, Folsom, CA, USA) was coupled with an electroantennographic detector (EAD; Syntech). The injector temperature was 250 °C. The oven was programmed at 120 °C for 1 min, increased to 250 °C at 5 °C/min, increased to 280 °C at 20 °C/min, and held at 280 °C for 5 min. The detector temperature was 300 °C. Nitrogen served as the carrier gas. Male antennae were excised with micro-scissors, the tips were removed, and antennae were mounted between two metal electrodes using conductive gel (Spectra 360, Parker Lab, Fairfield, NJ, USA). The electrode holder was inserted into the EAD probe, and testing began after a stable baseline was obtained. The GC effluent was split 1:1 between the EAD and FID. Five female equivalents of gland extract were used per GC-EAD run. Autospike V3.9 (Syntech) was used for data analysis.

#### 2.3.4. GC-MS

Pheromone gland extracts were analyzed using an Agilent 5977B MS system coupled to an Agilent 7820A GC equipped with the same HP-5 capillary column. The analytical method followed Jiang et al. [15] with minor modifications. The same temperature program as in GC-EAD was used. Samples were injected in splitless mode, and helium served as the carrier gas. Data were analyzed using the Windows NT/MASS Spectral Search Program (Version 1.7). Electron impact ionization was performed at 70 eV. The ion source and interface temperatures were set at 230 °C and 250 °C, respectively.

#### 2.3.5. GC-EAD Response of Gland Extracts and Standard Samples

The gland extraction and GC-EAD methods followed the procedures described above. Based on GC-EAD and GC-MS results, the standards E11-14Ac and Z11-14Ac were diluted to 0.5 μL/mL.

#### 2.3.6. Preparation of Sexual Attractants and Field Application

Lures were prepared using the identified pheromones E11-14Ac and Z11-14Ac. Controls included 7Z-12Ac and 9Z-12Ac. For each pheromone, 125 μL of compound and 0.25 g of Vaseline were added to a 1 mL centrifuge tube to create the lure mixture. Mixtures were stored at −20 °C.

Trapping tests were conducted in Huangjiazhai Village, Datong County, and Xiliangqi Village, Huangzhong County, Xining City, Qinghai Province (36°31′37.82″ N, 101°35′30.06″ E). Basins (20 cm × 10 cm × 8 cm) containing water with detergent were used, with lures placed 1 cm above the water level. Six traps were arranged per 666.67 m^2^ area, and insect numbers were recorded on days 3, 7, and 15.

## 3. Results

### 3.1. Distinguishing the Sex of E. extimalis Pupae

**Figure 1:**
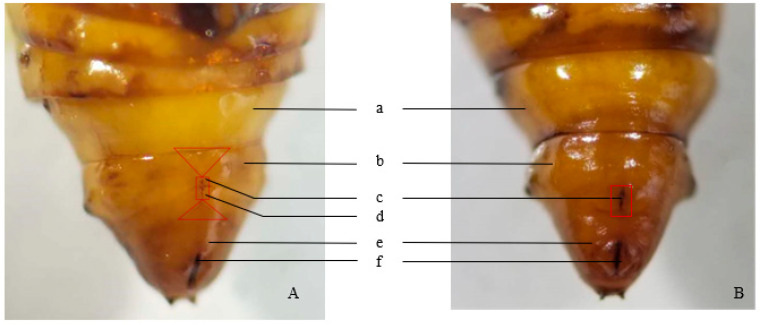
The abdominal end of *E. extimalis* pupae. (**A**) female; (**B**) male; a: 7th abdominal segment; b: 8th abdominal segment; c: gonopore; d: oviposition holes; e: 10th abdominal segment; f: anus. The two triangles, respectively, represent the Y-shaped protrusions in the 7th abdominal segment and the 9th abdominal segment. And the area within the rectangular frame indicates the longitudinal crack in the 8th abdominal segment in Figure 1.

Female pupae possess a longitudinal crack spanning the seventh and ninth abdominal segments on the upper central ventral surface of the eighth abdominal segment. The sides of this crack are open and flat, without semicircular protrusions. This crack forms a connection between the genital and oviposition pores. Two Y-shaped protrusions occur between the seventh and ninth abdominal segments, and the abdominal segmentation is not distinct (Figure 1A).

In male pupae, the eighth abdominal segment lacks a longitudinal crack; instead, a crack is present in the center of the ninth ventral segment, with semicircular tubercles on both sides (Figure 1B).

Sex was determined in 78 pupae based on the above morphological features (Figure 1), and each pupa was reared individually until eclosion. A total of 68 adults emerged (38 females and 30 males). Sex identification accuracy reached 100%, confirmed by examining adult abdominal and genital structures (Table 1).

**Figure 2:**
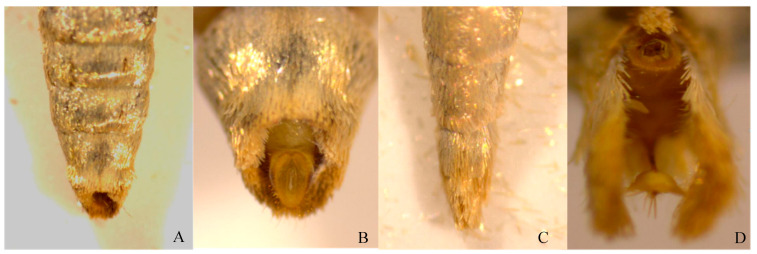
Ventral view of *E. extimalis* adults. (**A**) abdomen of female; (**B**) abdominal end of female; (**C**) abdomen of male; (**D**) abdominal end of male.

Under a dissecting microscope, the female adult abdomen appeared stout, and the abdominal end was covered with elliptical circular hairs, enclosing a pair of anal protrusions and oviposition valves. The oviposition pore was located at the anal protrusion (Figure 2A,B). In males, the abdominal tip was apiculate, with creamy-yellow scopulae and a pair of valvae (Figure 2C,D).

**Figure 3:**
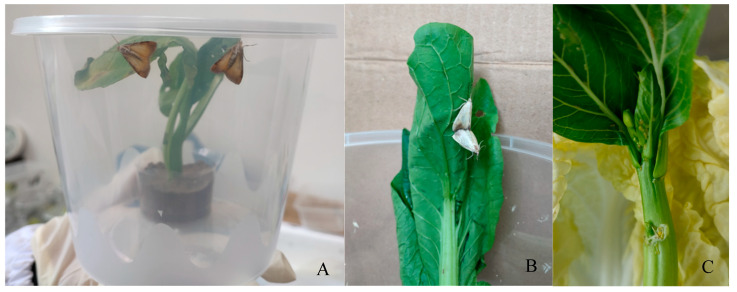
Mating and oviposition of *E. extimalis* adults. (**A**,**B**) mating adults; (**C**) oviposition by emerged adults.

To further validate sex determination, indoor mating and oviposition experiments were conducted. Among the 72 pupae, the eclosion rates for females and males were 86.11% and 80.56%, respectively (Table 2). A total of 29 mating pairs were observed (Figure 3A,B). All 29 pairs successfully completed mating and oviposition. Eggs were creamy yellow, forming herringbone-like clusters (Figure 3C).

These sex identification and validation procedures established a reliable foundation for subsequent isolation and identification of *E. extimalis* sex pheromones.

### 3.2. Identification of Sex Pheromone

#### 3.2.1. GC-EAD Analysis

**Figure 4:**
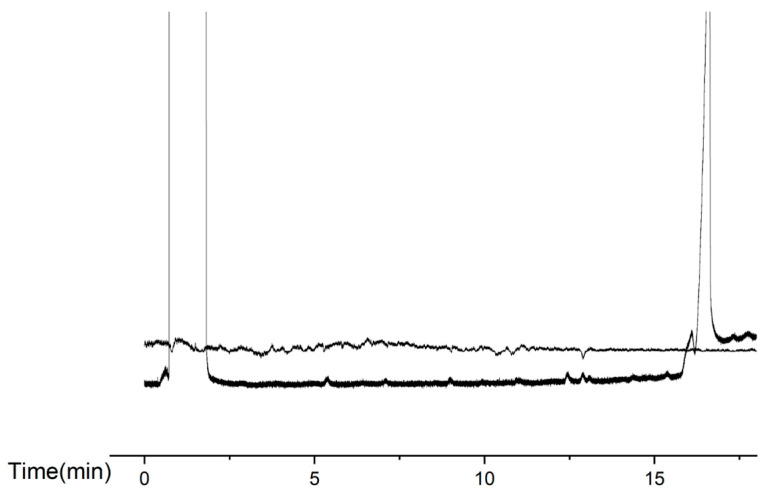
GC-EAD chromatograms of *E. extimalis* gland extracts. The GC retention times showing male responses: 7.13, 9.00, 9.97, 10.90, 12.03, 12.43, 12.90, and 13.11 min.

Male antennae responded to extracts from virgin females. Each antenna was stimulated up to three times. The male electroantennographic responses corresponded to GC retention times of 7.13, 9.00, 9.97, 10.90, 12.03, 12.43, 12.90, and 13.11 min. These results were consistent across five replicated GC-EAD runs (Figure 4).

In temporal comparisons of gland extract activity, extracts collected between 23:00 and 00:00 h exhibited markedly stronger EAG responses than those collected at other time intervals.

#### 3.2.2. Identification of Sex Compounds

**Figure 5:**
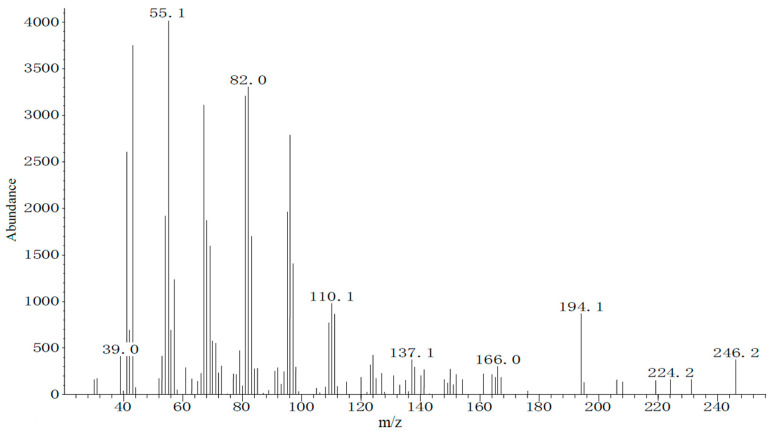
Mass spectrum of the female gland extract from unmated *E. extimalis* at retention time 13.11 min.

Chemical characterization of the female sex gland extract was performed via GC-MS using the same temperature program as in the GC-EAD analysis. Total ion chromatogram evaluation, combining molecular and fragment ion peaks, indicated that the pheromone-related peak corresponding to the GC-EAD response appeared at 13.11 min. The molecular ion (*m*/*z*) of this compound was 254 (Figure 5). These data suggest that the main pheromone components of *E. extimalis* females are E11-14Ac and Z11-14Ac.

**Figure 6:**
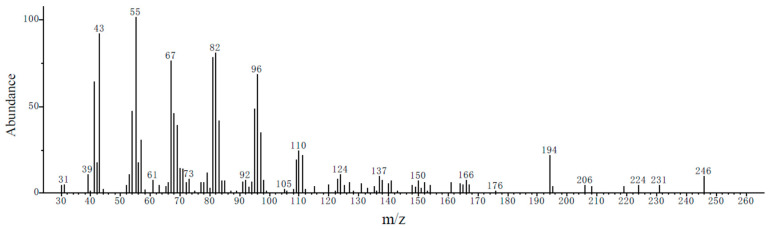
Mass spectra of the chemical standards E11-14Ac and Z11-14Ac at 13.11 min.

Analysis of authentic standards (E11-14Ac and Z11-14Ac) under identical conditions revealed mass spectra identical to those of the female gland extract (Figure 6).

### 3.3. GC-EAD Response Comparison of Standard Compounds and Sex Gland Extraction

**Figure 7:**
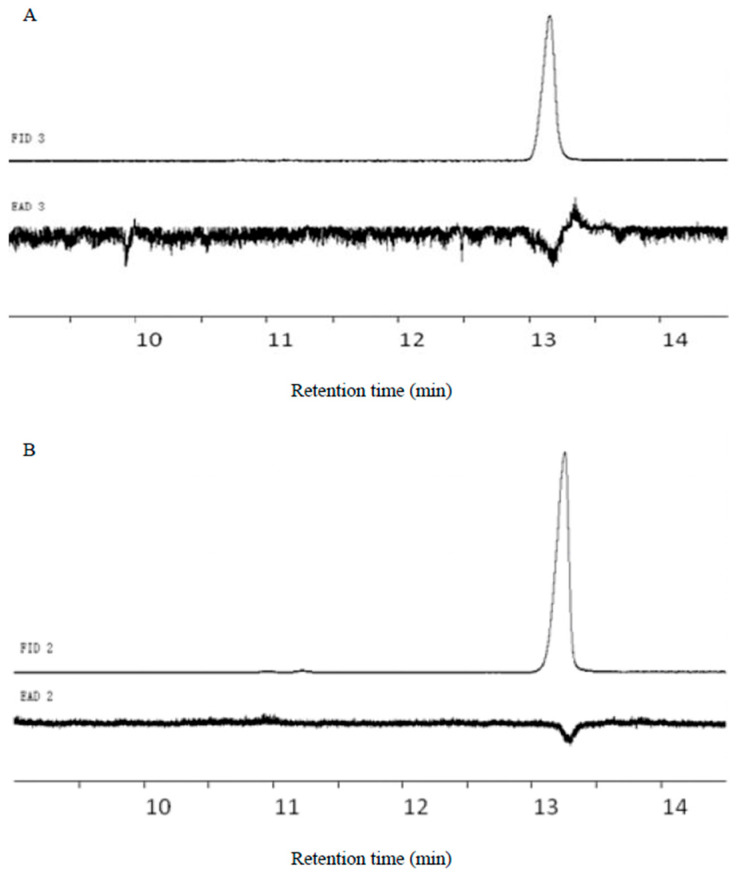
GC-EAD response profiles of pheromone standards. (**A**) E11-14Ac retention time 13.11 min; (**B**) Z 11-14Ac retention time 13.25 min.

E11-14Ac and Z11-14Ac elicited GC-EAD responses in males, with retention times of 13.11 min and 13.25 min, respectively (Figure 7).

**Figure 8:**
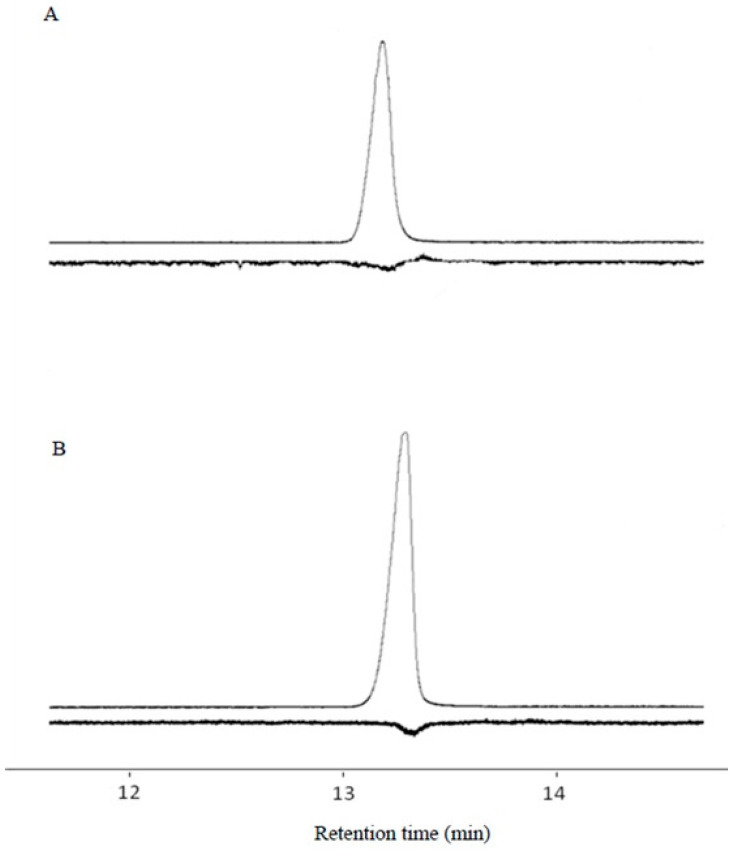
GC-EAD response profiles of sex gland extracts. (**A**) E11-14Ac retention time 13.11 min; (**B**) Z11-14Ac retention time 13.25 min.

The gland extract produced an EAD peak at 13.11 min (Figure 8).

These results indicate that E11-14Ac is likely the principal component of the *E. extimalis* sex pheromone.

### 3.4. Trapping Effect of Sex Lures in Fields

**Figure 9:**
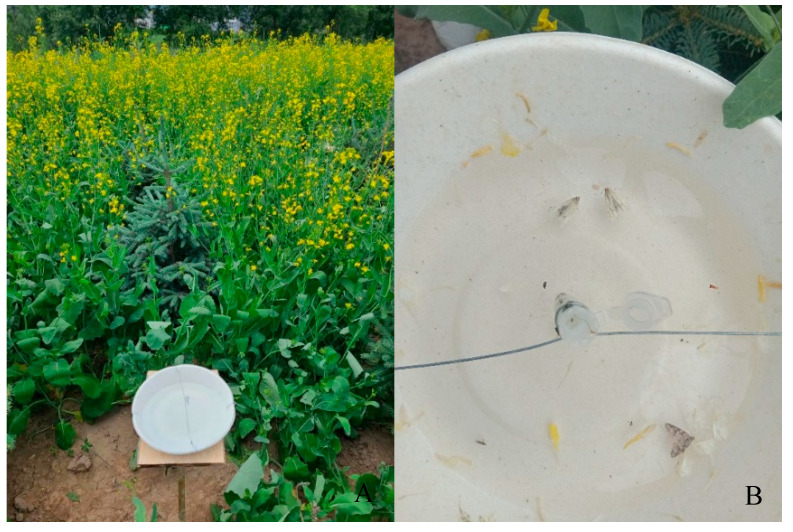
Trapping setup and captured adults. (**A**) Field protocol for deploying pheromone traps against the *E. extimalis*. (**B**) Male adults of *E. extimalis* collected in pheromone trapping.

**Figure 10:**
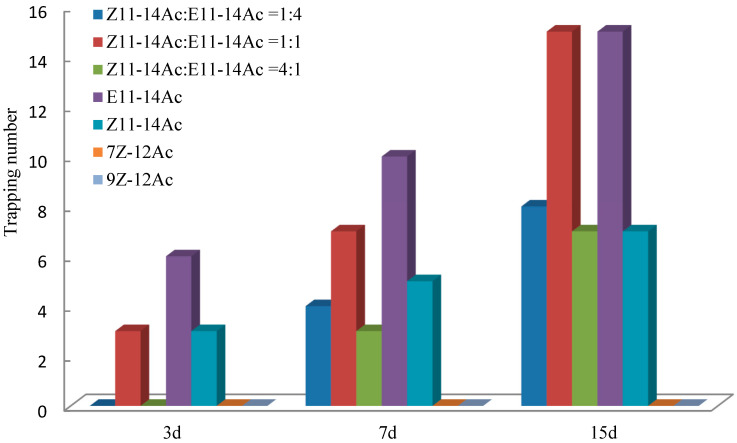
Trapping effects on male *E. extimalis* in Huangzhong. E11-14Ac trapped the most adults, followed by the 1:1 mixture.

In the Huangzhong test fields (Figure 9), most adults were captured using E11-14Ac, followed by the mixture Z11-14Ac:E11-14Ac = 1:1. On day 3, Z11-14Ac:E11-14Ac = 1:1 and Z11-14Ac alone yielded equal captures, whereas mixtures of 4:1 and 1:4 showed no trapping effect. By days 7 and 15, both mixtures (4:1 and 1:4) displayed clear activity, although they captured fewer males than the 1:1 ratio (Figure 10).

**Figure 11:**
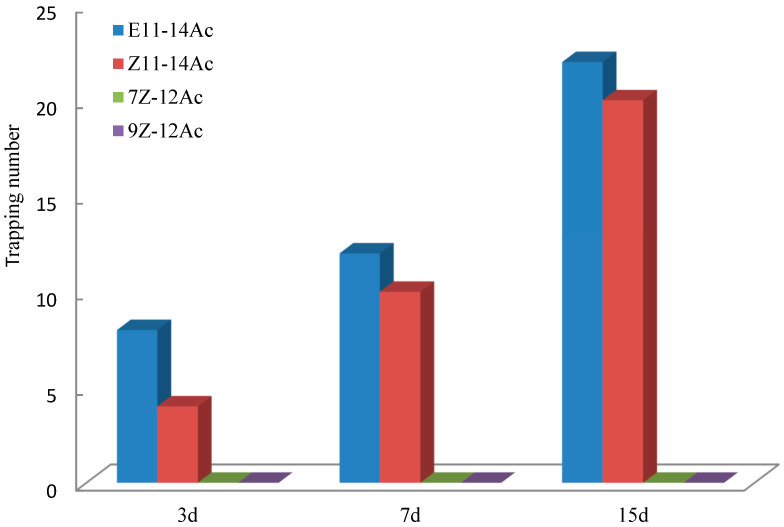
Trapping effects on *E. extimalis* male adults in Datong. The mixture attracted males, but E11-14Ac alone showed superior performance.

A mixture of Z11-14Ac and E11-14Ac attracted *E. extimalis*, although E11-14Ac alone produced the strongest effect (Figure 11). Controls (7Z-12Ac and 9Z-12Ac) showed no trapping effect in the Datong test.

Collectively, GC-EAD, GC-MS, and field trapping data indicate that E11-14Ac is the primary component of the *E. extimalis* sex pheromone.

## 4. Discussion

Sexual dimorphism has been documented in several Lepidoptera species, including *Phyciodes phaon*, *Pieris rapae*, *Scotogramma trifolii*, and *Ostrinia furnacalis* [16,17,18,19]. However, dedicated research on *E. extimalis* has been lacking. The pupal morphology of *E. extimalis* exhibits sexual dimorphism typical of Crambidae. Females possess an oviposition pore connected to the genital pore in the eighth abdominal segment, while males exhibit a genital crack at the ninth segment, accompanied by semicircular tubercles. Adult females have stout abdomens with elliptical circular hairs enclosing a pair of oviposition valvae. Male adults possess an apiculate abdominal tip with a pair of valvae [20].

GC-MS-EAD remains the standard method for identifying insect sex pheromones. Traditional preparations require antennal excision and electrode mounting, which compromises insect viability and may generate artifacts. While many pheromone components are clearly visible in chromatograms (e.g., *E. extimalis*), some species require structural confirmation through retention time comparison with authentic standards, as demonstrated for *Apolygus lucorum* (Zhang et al., 2011) [21].

The compositions of sex pheromones vary widely among genera and species within the Pyralidae (a family of Lepidoptera). For example, the primary component of the sex pheromone of *Evergestis forficalis* is E11-14Ac [22]. The main constituent of the sex pheromone of *E. pallidata* is 2-phenylacetaldehyde [23]. The principal components of the sex pheromone of *E. aenealis* are Z7-12Ac and Z9-14Ac (Toth et al., 1994) [24]. The major components of the sex pheromone of *E. frumentalis* are Z11-16Ald and Z9-14Ald [24]. Notably, for *E. extimalis* (the marbled yellow pebble moth)—a species sharing the same name as that studied here but belonging to a different strain—the primary sex pheromone components have been reported as Z9-14Ac and Z7-12Ac (3), although other reports indicate that Z9-14Ac alone is the main pheromone component [25]. The fennel-associated *E. extimalis* strain in China remains insufficiently studied. Our research demonstrates that E11-14Ac elicits activity in *E. extimalis*, whereas the role of Z11-14Ac requires further investigation. Z11-14Ac is a 14-carbon straight-chain lipid. Most Lepidoptera sex pheromones consist of 12–16 carbon straight-chain lipids with unsaturated olefinic [26,27]. Insect sex pheromones often contain multiple components, such as those of *Spodoptera frugiperda*, which include Z9-14Ac, Z7-12Ac, Z11-16Ac, and E7-12Ac [28]. However, some insects rely on a single pheromone component, such as *Loxostege sticticalis* (E11-14Ac) (Leal, 1998) [29] and *Ostrinia latipennis* (E11-14OH) [30]. The structure of E11-14Ac resembles that of the *Plutella xylostella* pheromone Z11-16Ac [31]. Structural differences also occur among insect pheromones. For instance, the pheromone compounds of *Trogoderma* spp. and *Glossina morsitans* possess side chains, whereas others feature heterocyclic structures [29]. Additionally, pheromone variation is influenced by geographic distribution, as seen in *Galleria mellonella* [32].

Pheromone activity varies with factors such as chemical structure [33], test method, and interference from additional components [26]. Furthermore, the number of ion peaks observed in mass spectrometry does not necessarily correspond to the number of active components. For example, *Andraca bipunctata* produces numerous ion peaks (41, 55, 67, 81, 95, 109, 123, 133, 151, 264 *m*/*z*), yet only one active ingredient—E11,E14-18Ald—was ultimately identified [34]. Thus, despite complex mass spectra, only a single active compound may be present [34].

The effectiveness of lure trapping is influenced by pheromone composition and dosage. For instance, the blend Z7-12Ac:Z9-14Ac:Z11-16Ac (3:1:1) increased *Agrotis ypsilon* captures by 2.8-fold compared with the 3:1 ratio alone [35]. Experiments using different doses of E11 and E14-18Ald showed that a 0.9 mg lure of E11,E14-18Ald produced the highest attraction to *A. bipunctata* [34]. Trap design also affects capture efficiency; large ship-shaped traps outperform triangular and small ship-shaped traps [36]. For *Spodoptera exigua*, silicone cap lures yield higher trapping efficiency than capillary lures [17].

Our research found that *E. extimalis* female pupae exhibit a longitudinal crack on the upper central ventral surface of the eighth abdominal segment, connecting the seventh and ninth segments. Male pupae lack this crack on the eighth segment but possess a longitudinal crack on the ninth ventral segment, with semicircular tubercles on each side. This study identifies E11-14Ac as the primary sex pheromone component of the Chinese *E. extimalis* strain, providing a scientific basis for pheromone-based pest management.

## Figures and Tables

**Table 1:** Identified results of male and female pupae.

Sex Identification Across Insect Developmental Stages	Female	Male
Number of pupae with sex determined	42	36
Number of emerged adults	38	30
Number of adults with sex identified	38	30

**Table 2:** Numbers of identified male and female pupae, emerged adults, and mating.

Sex Identification Across Insect Developmental Stages	Female	Male
Number of pupae with sex determined	36	36
Number of emerged adults	31	29
Number of mating adults	29	29

## Data Availability

Data are contained within the article.

## References

[1] Blomquist G.J., Vogt R.G. (2021). Insect Pheromone Biochemistry and Molecular Biology.

[2] Suckling D.M. (2015). Sex pheromones and semiochemicals offer an elegant future for pest management and biosecurity. Acta Hortic..

[3] Ando T., Yamamoto M. (2020). Semiochemicals containing lepidopteran sex pheromones: Wonderland for a natural product chemist. J. Pestic. Sci..

[4] Foster S.P., Anderson K.G. (2022). Some factors influencing calling behavior and mass emission rate of sex pheromone from the gland of the moth *Chloridea virescens*. J. Chem. Ecol..

[5] Levi-Zada A., Byers J.A. (2021). Circadian rhythms of insect pheromone titer, calling, emission, and response: A review. Sci. Nat..

[6] Jiang N.J., Mob T., Guo H., Yang J., Tang R., Wang C.Z. (2022). Revisiting the sex pheromone of the fall armyworm *Spodoptera frugiperda*, a new invasive pest in South China. Insect Sci..

[7] Sun F., Hu Y.Y., Du J.W. (2002). The sex pheromone communication system of *Spodoptera litura* (Fabricius). Acta Entomol. Sin..

[8] Nesbitt B.F., Beevor P.S., Hall D.R., Lester R. (1979). Female sex pheromone components of the cotton bollworm, *Heliothis armigera*. J. Insect Physiol..

[9] Yan J.J., Zhang M., Ali A., Du X., Mei X., Gao Y. (2022). Optimization and field evaluation of sex-pheromone of potato tuber moth, *Phthorimaea operculella* Zeller (Lepidoptera: Gelechiidae). Pest Manag. Sci..

[10] Wang L.Y., Yang C.X., Guo B.B., She D.G., Mei X.G., Yang X.L., Ning J. (2022). Research progress and application prospects on insect sex pheromone. Chin. J. Pestic. Sci..

[11] Liu W.C., Liu Z.D., Zhu X.M., Du Y.J. (2022). Development and Application of Insect Sex Pheromone Technology in China. Chin. J. Biol. Control.

[12] Wang Y.M. (2017). Studies on Pesticide-Resistance of Evergestis extimalis Scopoli Populations to Chlorpyrifos and Beta-Cypermethrin.

[13] Qin M.G., Lal Y.P. (2022). Sexsual identification of *Evergestis extimalis* Seopoli (*Lepidoptera pyralidida*). J. Qinghai Normal Univ. (Nat. Sci.).

[14] Huang C.H., Li J.J., Zhou L., Yan F.-M. (2014). EAG and GC-EAD techniques. Chin. J. Appl. Entomol..

[15] Jiang N.J., Tang R., Wu H., Xu M., Ning C., Huang L.Q., Wang C.Z. (2019). Dissecting sex pheromone communication of *Mythimna separata* (Walker) in North China from receptor molecules and antennal lobes to behavior. Insect Biochem. Mol. Biol..

[16] Genc H. (2005). Determination of sex in pupae of *Phyciodes phaon* (Lepidoptera: Nymphalidae). Fla. Entomol..

[17] Chen Z.L., Yang X.L., Zhang Z.N. (2010). A method used for distinquishing between the sexes of Pieris rapae pupae. Chin. J. Appl. Entomol..

[18] Zhao Q., Zhang Y.H., Liu H. (2012). A method used for distinguishing between the sexes of *Scotogramma trifolii*. Chin. J. Appl. Entomol..

[19] Zhang J., Du X., Wang Z.Y. (2013). A method for the rapid sex-determination of pupae of the Asian corn borer, *Ostrinia furnacalis*. Chin. J. Appl. Entomol..

[20] Li D.W., Wu Y.J., Jiang X.J. (2008). A method for identifying the sex of *Lasiognatha cellifera* larva, pupa and adult. Chin. J. Appl. Entomol..

[21] Zhang T. (2011). The Study of the Extraction, Identification and Application of Sex Pheromone Produced by Apolygus Lucorum.

[22] Hall D.R., Read J.S. (1979). Sex attractants for two zygaenid moths. J. Entomol. Soc. S. Afr..

[23] Cantelo W.W., Jacobsen M., Hartstack A.W. (1982). Moth trap performance: Jackson trap vs. Texas pheromone trap. Southwest. Entomol..

[24] Toth M., Szücs G., Szirüki G. (1994). Male sex attractants for some Lepidopterous pests of the genera Evergestis and Pyrausta (Lepidoptera: Pyralidae). Acta Phytopathol. Entomol. Hung..

[25] Konstantin E.N., Vorotyntseva A.F., Rastegaeva V.M., Pynzar`s B.V., Drozdov Y.I., Boubetryn I.N., Kovalev B.G. (1980). Field attractancy of synthetic compounds for males of some Lepidoptera. Novye Met. Zasch. Rast. USSR.

[26] Yan F.M. (2011). Chemical Ecology.

[27] Nesbitt B.F., Beevor P.S., Hall D.R., Lester R., Dyck V.A. (1975). Identification of the female sex pheromones of the moth, *Chilo suppressalis*. J. Insect Physiol..

[28] Li B. (2021). Component Analysis and Application Technology of Sex Pheromone of Spodoptera frugiperda.

[29] Leal W.S. (1998). Chemical ecology of phytophagous scarab beetles. Annu. Rev. Entomol..

[30] Mori K. (1996). Molecular asymmetry and pheromone science. Biosci. Biotech. Biochem..

[31] Wang X.P., Fang Y.L., Zhang Z.G. (2003). Research on diamondback moth sex pheromone and its applications. Plant Prot..

[32] Leyrer R.L., Monroe R.E. (1973). Isolation and identification of the scent of the moth, *Galleria mellonella*, and a revaluation of its sex pheromone. J. Insect Physiol..

[33] Silk P.J., Tan S.H., Wiesner C.J. (1980). Sex Pheromone chemistry of the eastern spruce budworm. Environ. Entomol..

[34] Cui S.W., Zhao D.X., Zhang J.X. (2022). Sex Pheromone of Andraca bipunctata Mainland Population in China: Identification and Population Monitoring. J. Tea Sci..

[35] Xiang Y.Y. (2007). Extraction and Identification of the Black Cutworm’s Sexpheromone and Correlative Studies on Its Biology.

[36] Wang S.M., Gao C.G., Liang S.J. (2018). Effect of different traps on the insect trapping of sex attractant of *Dendrolimus tabulaeformis*. J. Environ. Entomol..

